# Hydroalcoholic extract of *Crocus sativus* effects on bronchial inflammatory cells in ovalbumin sensitized rats

**Published:** 2013

**Authors:** Maryam Mahmoudabady, Ali Neamati, Somayyeh Vosooghi, Heydar Aghababa

**Affiliations:** 1 *Applied Physiology Research Centerand Department of Physiology, School of Medicine, Mashhad University of Medical Sciences, Mashhad, I. R. Iran*; 2 *Department of Biology, Faculty of Science, Mashhad Branch, Islamic Azad University, Mashhad, I. R. Iran*; 3 *Department of Biology, Payame Noor University, Boshruyeh, South Khorasan, I. R. Iran*; 4 *Department of Biology, Faculty of Science, Arsanjan Branch, Islamic Azad University, I. R. Iran*

**Keywords:** Asthma, *Crocus sativus*, Inﬂammation, Ovalbumin, Rat, Sensitization, WBC

## Abstract

**Objective:** Inflammation is one of the major components of asthma**.** Our aim was to evaluate the effects of *C. sativus* extract on total and differential white blood cells (WBC) count in lung lavage fluid (LLF) of ovalbumin-sensitized rats.

**Materials and Methods:** Forty rats were divided into five groups (*n* = 8 for each group) as control (C), sensitized with injection and inhalation of ovalbumin (OA) alone (S), and three groups of sensitized and treated with different doses of *C. sativus* extract (S50EX, S100EX, and S200EX groups). Total and differential WBC counts of LLF were evaluated in control, sensitized, and treated sensitized groups.

**Results:** Total WBC count, neutrophil, and eosinophil percentage in LLF were increased in sensitized animals compared with the control group (p0.001). Treatment of sensitized animals with all doses of the extract significantly reduced WBC number and the percentage of neutrophil and eosinophil compared with the sensitized animals (p0.01–0.001).

**Conclusion:** According to these results, the extract of *C. sativus* could be effective on alleviating lung inflammatory cells specially eosinophils in lung lavage of sensitized animals which may indicate a preventive effect of this plant on lung inflammation in asthma.

## Introduction

Asthma is a chronic inflammatory disease of the respiratory tract that causes bronchial hyperresponsiveness and airway remodeling (Mauad et al., 2007 ▶). Several factors influence the disease, for example, genetic (e.g., a history of asthma in the family, atopy), environmental (e.g., air pollution, occupational exposure, viral infections, allergen exposure), and life style (e.g., smoking habits, food) (Bloemen et al., 2007 ▶). Airway remodeling refers to changes in the airway structure and is characterized by submucosal gland enlargement, neovascularization, epithelial alterations, basement membrane fibrosis, fibroblast and smooth muscle cell hypertrophy, and hyperplasia (Bergeron et al., 2007 ▶). Exposure to inhaled allergens such as pollen, dust mites, mold, or animal dander can initiate an acute immune response in allergen-sensitive individuals that lead to airway inflammation (Agrawal and Shao, 2010 ▶). Asthmatic airways are infiltrated with inflammatory cells consisting of eosinophils, lymphocytes, neutrophils, mast cells, and phagocytes (Bradley et al., 1991 ▶; Haley et al., 1998 ▶). Inflammatory mediators released by these cells such as cytokines and growth factors are the effectors of chronic inflammation (Ishmael, 2011 ▶).

In susceptible individuals, the inflammation causes recurrent episodes of wheezing, breathlessness, chest tightness, and cough particularly at night and/or early morning (Skrepnek and Skrepnek, 2004 ▶). Recent research implies the role of antioxidant therapy in asthma (1992). Crocus sativus L. (saffron) is a flowering plant belonging to the Iridaceae (Hosseinzadeh and Nassiri-Asl, 2012 ▶). Main components of the stigma of this plant include crocins, safranal, picrocrocin, ketoisophorone, isophorone, and glycosidic terpenoids (Tarantilis et al., 1995 ▶).

In traditional medicine, antispasmodic and expectorant effects of saffron has been mentioned (Rios et al., 1996 ▶). Pharmacological studies have proved that *C. sativus* extracts or its constituents have radical scavenging and antioxidant properties (Assimopoulou et al., 2005a ▶; Rios et al., 1996 ▶; Verma and Bordia, 1998 ▶). Antitussive (Hosseinzadeh and Ghenaati, 2006 ▶) and anti-inflammatory (Boskabady et al., 2012 ▶; Hosseinzadeh and Younesi, 2002 ▶; Mousavi and Bathaie, 2011 ▶) effects of *C. sativus* as well as its relaxant effect on tracheal smooth muscle have also been reported (Boskabady and Aslani, 2006 ▶). Previous studies showed the inhibitory effect of the plant on histamine (H_1_) receptor (Boskabady et al., 2010b ▶) and its stimulatory effect on -adrenoceptors (Nemati et al., 2008 ▶).

Therefore, according to the above-mentioned properties of *C. sativus* and the role of inflammation in pathogenesis of asthma, the aim of this study was to investigate the effect of the hydroalcoholic extract of *C. sativus* on total and differential white blood cells (WBC) count in lung lavage of ovalbumin-sensitized rats. 

## Materials and Methods

**Plant material and preparation of the extract**

C. sativus was harvested from saffron farms of Boshrooyeh (northeast of Iran) and the plant was identified by botanists in the herbarium of Ferdowsi University of Mashhad and the specimen number of the plant is 293-0303-1. Hydroalcoholic extract was prepared with 10 g of its ground petal stigma and 400 mL of 70% aqueous-alcohol solution in a Soxhlet extractor for 14 h. The prepared extract was concentrated to 100 mL with a rotatory evaporator in low pressure and filtered through a 0.2-mm filter to be sterilized. The resulting extract was concentrated under reduced pressure and stored at -20 °C until being used. The extract was dissolved in saline in order to arrive at desired doses and was then applied.

**Animal sensitization and animal groups**

Forty male Wistar rats weighing approximately 200–250 g were housed in Plexiglas cages in a temperature-controlled environment (20±2 °C) on a 12-h light-dark schedule (lights on from 6:00 am to 6:00 pm) with free access to water and rodent chow. The animals were acclimatized for at least 7 days before use in experiments and then were sensitized according the method described previously (Boskabady and Adel-Kardan, 1999; Boskabady and Ziaei, 2003). Briefly, rats were sensitized to 1 mg ovalbumin (OA) (Sigma Chemical Ltd, UK) and 50 mg Al(OH)3 dissolved in 0.5 ml saline intraperitoneal (i.p.). One week later, they were given 0.02 mg OA and 50 mg Al(OH)3 dissolved in 0.5 ml saline i.p. as a booster dose. From day 14, sensitized animals were exposed to an aerosol of 4% OA for 18±1 days, 5 min daily.

 The aerosol was administered in a closed chamber, dimensions 30×20×20 cm3 using a nebulizer (CX3, Omron Healthcare Europe B. V., Netherlands). Control animals were treated similarly, but saline was used instead of OA solution. Treated animals received different doses of C. sativus extract twice a week as intraperitoneal injection simultaneously to sensitization for 32 days. The study was approved by the ethics committee of Islamic Azad University, Mashhad branch.

The animals were randomly divided into five groups (*n* = 8 for each group) as follows:

1. Control group (not sensitized, group C)
2. Sensitized with ovalbumin (OA) alone (S)
3. Sensitized and treated with 50 mg/kg extract of* C. sativus* post sensitization during 32 days (S50EX)
4. Sensitized and treated with 100 mg/kg extract of* C. sativus* post sensitization during 32 days (S100EX)
5. Sensitized and treated with 200 mg/kg extract of* C. sativus* post sensitization during 32 days (S200EX)

**Lung lavage and its WBCs count**

At the end of the experiment period, rats were anesthetized by i.p. injection of chloral hydrate (400 mg/kg). After opening the chest, trachea and lungs were dissected and external surfaces were washed with distilled water then the trachea was cannulated and the lungs were lavaged with 2 mL of phosphate buffer saline (PBS) buffer five times (total=10 mL). One milliliter of lung lavage fluid (LLF) were stained with Turk solution and counted in duplicate in a hemocytometer (in a Burker chamber). The Turk solution consisted of 1 mL of glacial acetic acid, 1 mL of gentian violet solution 1%, and 100 mL distilled water. The remaining LLF was centrifuged at 2,500   g at 4 C for 10 min. The supernatant was removed and the smear was prepared from the cells and stained with Wright-Giemsa. According to staining and morphological criteria, differential cell analysis was carried out under a light microscope by counting 100 cells and the percentage of each cell type was calculated.

**Statistical analysis**

All data are expressed as mean±standard error of the mean (SEM). Comparisons were performed using one-way analysis of variance (ANOVA) with SPSS software. *P*-values less than 0.05 were considered to be statistically signiﬁcant.

## Results

**Total WBC count of lung lavage**

**Figure 1:**
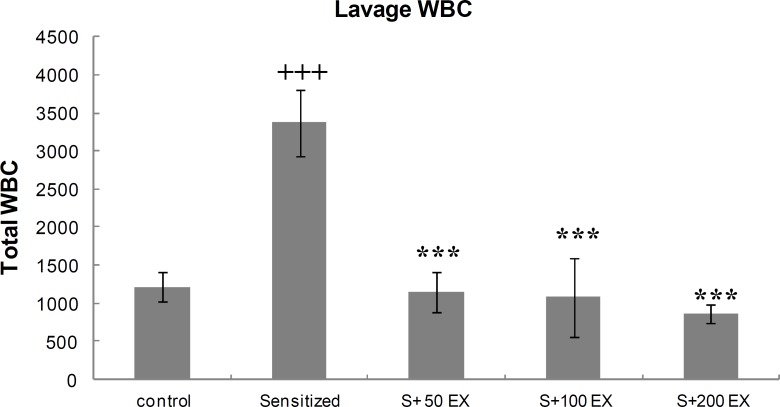
Total white blood cells (WBC) number (mean±standard error of mean (SEM) values) in lung lavage fluid (LLF) of control, sensitized (S), and three doses of extract (S EX). For each group, *n*=8.    p 0.001 versus control group. ***p 0.001 versus sensitized group

Figure 1 shows the effects of different doses of *C.* *sativus* extract on total LLF cell count in control, sensitized, and treated rats. Total WBC count in lung lavage of sensitized group was signiﬁcantly higher than those of control group (<0.001). In sensitized animals treated with different doses of *C. sativus*, total WBC decreased compared with sensitized group (p<0.001 for all groups).

**Differential** **WBC count of lung lavage**

**Figure 2:**
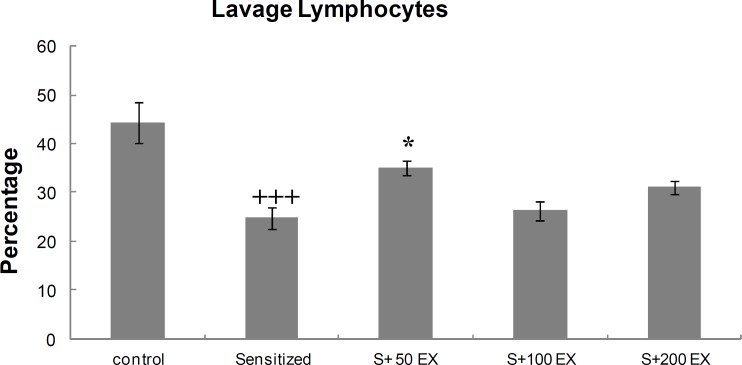
Percentage (mean±SEM values) of lymphocytes in LLF of control, sensitized (S), and three doses of extract (S EX). For each group, *n*=8.    p 0.001 versus control group. *p 0.05 versus sensitized group

<group S50EX significantly increased compared with the sensitized group (<). However, in two other groups of treatment, these increases were not significant (Figure 2).

**Figure 3:**
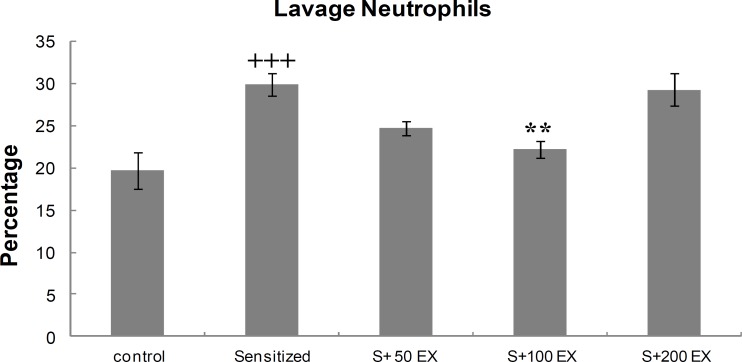
Percentage (mean±SEM values) of neutrophils in LLF of control, sensitized (S), and three doses of extract (S EX). For each group, *n* = 8.    p 0.001 versus control group. **p 0.01 versus sensitized group

<group S100EX compared with the sensitized group < (Figure 3).

**Figure 4:**
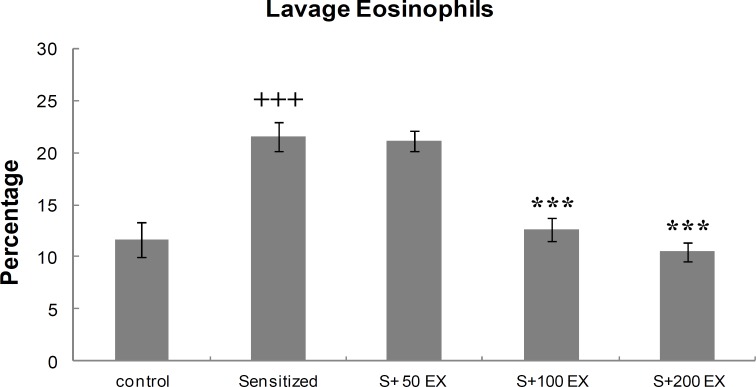
Percentage (mean±SEM values) of eosinophils in LLF of control, sensitized (S), and three doses of extract (S EX). For each group, *n* = 8.    p 0.001 versus control group. ***p 0.001 versus sensitized group

<S100EX and S200EX groups compared with the sensitized group (<Figure 4).

## Discussion

The results of the present study showed increased inflammatory cells especially eosinophil count in lung lavage in sensitized animals. It is well-documented that lung inflammation is the main characteristic of asthma (Mauad et al., 2007 ▶). 

Increased inflammatory cells in asthmatic patients (Bradley et al., 1991 ▶; Haley et al., 1998 ▶) and sensitized animals (Keyhanmanesh et al., 2010 ▶) have already been shown. Since airway inﬂammation is the pathological characteristic trait of asthma and many inﬂammatory cells play a role in this phenomenon (Busse et al., 1995 ▶; Kelly et al., 1988 ▶; Vosooghi et al., 2013 ▶) and according to our findings which showed increased total WBC, neutrophil and eosinophil count in lung lavage of sensitized animals, similar to the reports of previous studies (Boskabady et al., 2010a ▶; Boskabady et al., 2010b ▶; Busse et al., 1995 ▶), these results can confirm sensitization of rats in the present study. However, treatment of sensitized animals with extract of *C. sativus* leads to significant reduction in total and differential inflammatory cells. These findings indicate the suppression effect of the extract on lung inflammation of sensitized animals. 

While the main pathophysiological change of asthma is lung inflammation, the main therapy of this disease should be focused on suppression of this phenomenon. In this study, we observed possible inhibitory effect of extract on lung inflammation of sensitized animals. In fact, previous study showed the inhibitory effect of the extract of *C. sativus* on total and differential whole blood cells count. The effect of the plant on T helper 1/T helper 2 (Th_1_/Th_2)_ towards increased activity of Th_1_ and decreased activity of Th_2_ was also shown. The results of these studies support the findings of the present study (Bayrami and Boskabady, 2012 ▶; Boskabady et al., 2011b ▶). 

As mentioned in the ancient Persian medical books, *C. sativus* has a therapeutic effect on respiratory diseases (Avicenna, 1997 ▶; Mir Heidar, 2004 ▶; Zargari, 1990 ▶). Formerly, the relaxant effect of this plant and particularly its effective constituent, safranal on the tracheal smooth muscle was shown (Boskabady and Aslani, 2006 ▶). The inhibitory effect of the plant on histamine receptor (Boskabady et al., 2010b ▶) and its stimulatory effect on adrenoceptors have also been shown (Nemati et al., 2008 ▶). In a study, Hosseinzadeh *et al*. reported antitussive effect of *C. sativus* stigma, petal extracts, and its components, safranal, and crocin in guinea pigs (Hosseinzadeh and Ghenaati, 2006 ▶). Another study showed that *C. sativus* constituents such as crocin, crocetin, and safranal had free radicals scavenger and antioxidant effects (Assimopoulou et al., 2005b ▶). Moreover, anti-inflammatory effects of *C. sativus* stigma and petals extract has been tested in mice (Hosseinzadeh and Younesi, 2002 ▶).

Anti-inflammatory drugs which are used in treatment of asthma disease reduce airway inﬂammation, the main pathological feature of this disease. It is expected that the *C. sativus *extract reduces airway hyper-responsiveness which is distinct physiological abnormality in asthma by reducing lung inflammation because the plant leads to reduction in total inflammatory cells and eosinophils in sensitized animals.

The preventive effect of other plants such as *Nigella sativa *(Boskabady et al., 2011a ▶), carvacrol, the main constituent of *Zataria multiflora *(Jalali et al ▶.), and *Brassica napus *(Kabiri rad et al., 2013 ▶) on lung inflammation in sensitized guinea pigs and rats were also shown. Therefore, further studies including clinical trials needed to examine the therapeutic effect of these plants on asthma. However, it should be mentioned while the effect of *C. sativus *on lung inflammation of ovalbumin sensitized guinea pigs was previously reported (Jalali et al., 2013 ▶), but according to our study, it is the first time that this effect is shown in sensitized rat model.

The findings of the present study and those of previous studies suggest that *C. sativus *may have therapeutic effects on asthma by both bronchodilation and preventive effect on lung inflammation. 

In conclusion, the results of this study showed that the extract of *C. sativus* causes reduction of inflammatory cells, specially eosinophils, in lung lavage of sensitized animals which may indicate a preventive effect of this plant on lung inflammation in asthma.
